# Engineering Ultraporous and Highly Stable Polyacrylonitrile/Poly(vinyl alcohol) Sponges with High Water Absorption Capacity

**DOI:** 10.1021/acsami.5c18694

**Published:** 2025-12-16

**Authors:** Michèle-Louise Regner, Mateus Gruener Lima, Annika Thormann, Rafaela Debastiani, Juliana Martins de Souza e Silva

**Affiliations:** † Institute of Physics, 229897 Martin Luther University Halle-Wittenberg, 06120 Halle (Saale), Germany; ‡ Fraunhofer Institute for Microstructure of Materials and Systems IMWS, 06120 Halle (Saale), Germany; § Applied Nuclear Physics Research Group, 37894 State University of Londrina, 86057-970 Londrina, Brazil; ∥ Institute of Nanotechnology (INT), 150232 Karlsruhe Institute of Technology (KIT), 76344 Eggenstein-Leopoldshafen, Germany; ⊥ Karlsruhe Nano Micro Facility (KNMFi), Karlsruhe Institute of Technology (KIT), 76344 Eggenstein-Leopoldshafen, Germany

**Keywords:** hydrophilic ultraporous sponges, PVA/PAN sponges, micro-CT, nano-CT, *in situ* tensile test, digital volume correlation

## Abstract

Ultraporous sponges capable of absorbing large quantities of water-based liquids are of great interest in various fields of research. In this study, ultraporous polyacrylonitrile/poly­(vinyl alcohol) (PAN/PVA) sponges with exceptional water absorption capacity—up to 16000% of their dry weight—were produced through a four-stage process: electrospinning, short fiber suspension creation, freeze-drying, and PVA cross-linking with different maleic anhydride (MA) concentrations. Characterization by electron microscopy and X-ray microscopy revealed a porosity of 90% and an average fiber thickness of 0.4 μm. Mechanical tests demonstrate that the wet sponge is more compliant and experiences greater elongation than the dry sponge, with failure occurring below 20% strain in dry samples and above 40% when wet. *In situ* tensile testing in a micro-CT scanner and digital volume correlation analysis reveal significant morphological changes during stretching, including strain localization and microstructural variations. These findings provide insights into the mechanical behavior of the PAN/PVA sponges and identify regions that are prone to fracture. By the combination of electrospun PAN and PVA fibers in a stabilized ultraporous architecture, this work introduces a practical and efficient strategy for producing lightweight hydrophilic sponges for applications in water management, biomedical devices, and tissue engineering.

## Graphical abstract

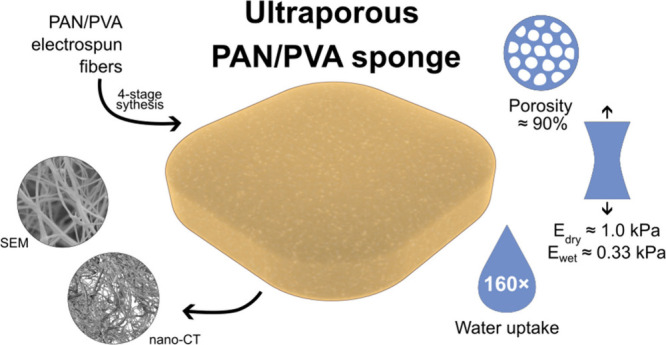

## 1 Introduction

Three-dimensional (3D) ultraporous polymeric materials have gained significant attention due to their remarkable porosity and elasticity. These unique properties make them suitable for a wide range of applications, including absorbents, oil/water separation systems, and diverse biomedical applications.^1,2^ In the medical field, these polymeric porous materials play an essential role as antibacterial wound dressings,^3^ polymer scaffolds for tissue engineering,^1^ and drug delivery systems.^2,4,5^

The fabrication of polymeric porous materials involves a variety of innovative methods such as 3D printing, foaming agents, and nanofibril assembly by Hofmeister effect.^6^ These techniques use the unique properties of polymers to create structures with tunable porosity and tailored functionality.^7^ Among these approaches, electrospinning remains a versatile and widely adopted method. It uses an electrostatic field to produce fibers with a high surface area and porosity, enabling the construction of 3D nanofibrous macrostructures.^8^ Several strategies for electrospinning 3D structures have been developed,^8^ with the choice of approach depending on factors such as the type of polymer, desired pore size, and specific application requirements, such as mechanical strength or biocompatibility.

In applications that involve direct contact with the human body in humid environments—such as wound dressings^9^ and biomedical implants^10,11^ —materials must exhibit sufficient mechanical stability during handling^12–14^ to ensure reliable performance. Moreover, they require high porosity for the successful cell growth,^15^ as well as significant hydrophilicity and water retention capabilities, allowing them to mimic the extracellular matrix (ECM), which supports cell growth and promotes tissue regeneration by maintaining a hydrated environment.^16^ However, achieving a balance between high porosity and mechanical robustness remains a significant challenge, particularly in applications where exposure to moisture is unavoidable.^4,5^ To address these challenges, numerous strategies have been explored, such as applying surface coatings,^1^ incorporating coadditives,^17^ or adjusting material density.^18^ One promising strategy involves integrating materials with complementary properties to achieve synergistic effects. For instance, poly­(vinyl alcohol) (PVA) is a hydrophilic polymer widely used in sponge fabrication due to its biodegradability, suitability for extended-release applications^14^ and its ability to facilitate cell attachment and growth, crucial for supporting tissue engineering structures.^19^ These properties make PVA particularly effective in applications involving water-based substances or humid environments,^5,20^ where maintaining a wet medium is crucial for optimal performance. However, the mechanical strength of PVA-based materials is often insufficient for demanding biomedical applications, which can limit their long-term stability.^21,22^ To address this limitation, materials with superior mechanical properties, such as polyacrylonitrile (PAN), can be introduced. PAN is a versatile polymer exhibiting multifunctional characteristics, including high chemical resistance and mechanical strength,^12,23–25^ as well as broad applicability in water management, purification, and separation technologies.^26–28^ However, its inherently hydrophobic nature restricts the use in applications that require water-absorbing properties.^29^

Recent studies have demonstrated the use of PAN nanofiber sponges, fabricated via electrospinning followed by freeze-drying, for the adsorption of organic compounds.^30,31^ In addition, several works have explored the cross-linking of sponges of various compositions with PVA to enhance their functionality for different applications, including separation of mixtures,^32^ such as oil–water systems,^33,34^ as well as biomedical uses^3,20^ and other industrial purposes.^35,36^ Combining PAN and PVA enables one to benefit from the multifunctionality of both materials, combining the high porosity and water absorptivity of PVA with the enhanced mechanical stability of PAN. This enables applications such as wound dressings, tissue engineering scaffolds, and high-capacity absorbents for water management, which address the challenge of balancing porosity, wet mechanical strength, and water retention.

In this work, we present for the first time lightweight, 3D, ultraporous, and mechanically stable hydrophilic polymeric sponges obtained by combining PVA and PAN. The sponges were prepared from the colloidal dispersion of short electrospun PVA/PAN thin fibers^15,37^ whose strength and hydrophilicity are, respectively, inherited from PAN and PVA. To overcome the water solubility of unmodified PVA,^38,39^ we used maleic anhydride (MA) to promote PVA cross-linking, thereby preserving the fibrous network skeleton^37^ and ensuring sponge stability.^40^ By adjusting the MA concentration, we aimed to fine-tune the stability and water absorption capacity of the sponges, thus addressing the challenges of maintaining both porosity and mechanical stability in light 3D ultraporous hydrophilic sponges. Comprehensive characterization using Fourier-transform infrared spectroscopy (FTIR), Soxhlet extraction followed by gravimetric assay, scanning electron microscopy (SEM), and micro- and nano-computed tomography (CT) confirms successful cross-linking and reveals an open-cell architecture with interconnected pores. *Ex-situ* and *in situ* tensile testing in a micro-CT setup enabled digital volume correlation (DVC) analysis^41^ to map strain-induced microstructural evolution. The resulting material is particularly suited for single-use applications such as wound dressings, drug delivery matrices, and disposable protective equipment, where immediate effectiveness, sterility, safe disposal, and short-term mechanical robustness are essential. This approach offers a promising solution for various biomedical and industrial needs.

## 2 Results

The synthesis of PAN/PVA sponges began with the production of electrospun fiber mats from PAN and PVA solutions (Figure 1). The resulting electrospun mat was cut into smaller pieces and placed in an isopropanol-water solution and further processed into a short fiber suspension. Maleic anhydride (MA) was added, and the suspension was frozen, followed by freeze-drying and heat treatment. Post-freeze-drying sponges measured 11.5 cm × 11.5 cm × 1.0 cm (Figure 2a), weighed 295 mg, and had a density of 2.23 ± 0.11 mg/cm^3^, within the typical ranges found in the literature.^15,42^ After cross-linking with MA, the sponges shrink, resulting in a decreased volume (10.5 cm × 10.5 cm × 0.5 cm) and a decreased weight (262 mg) but an increased density (4.75 ± 0.48 mg/cm^3^) (Figure 2b).

**Figure 1:**
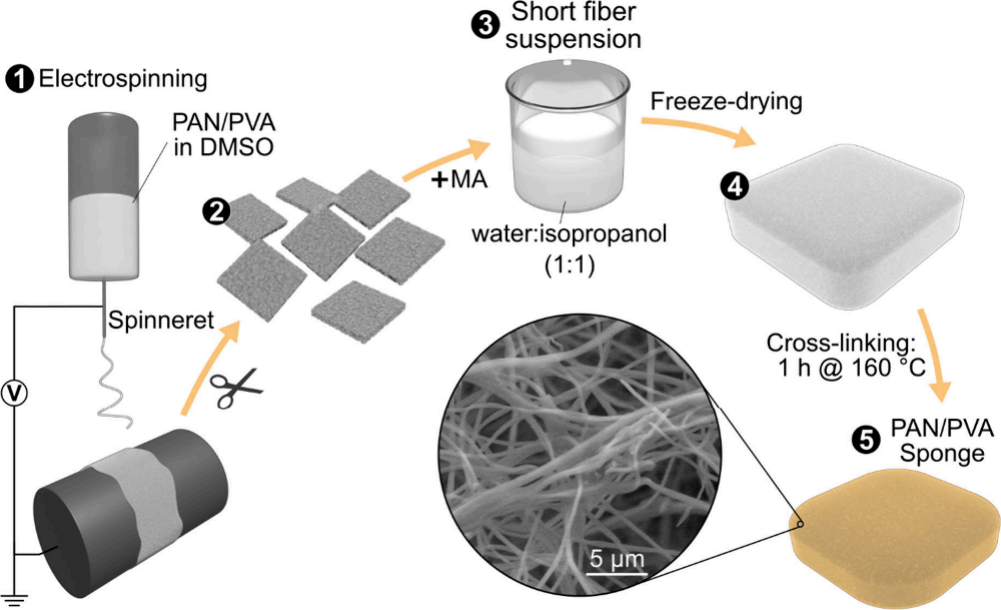
Steps for the synthesis of PAN/PVA sponges. First, a PAN/PVA electrospinning mat is formed, followed by the preparation of a suspension of short fibers by cutting the mat into small pieces and adding maleic anhydride (MA), freeze-drying it, and heat treatment to produce a stable sponge.

**Figure 2:**
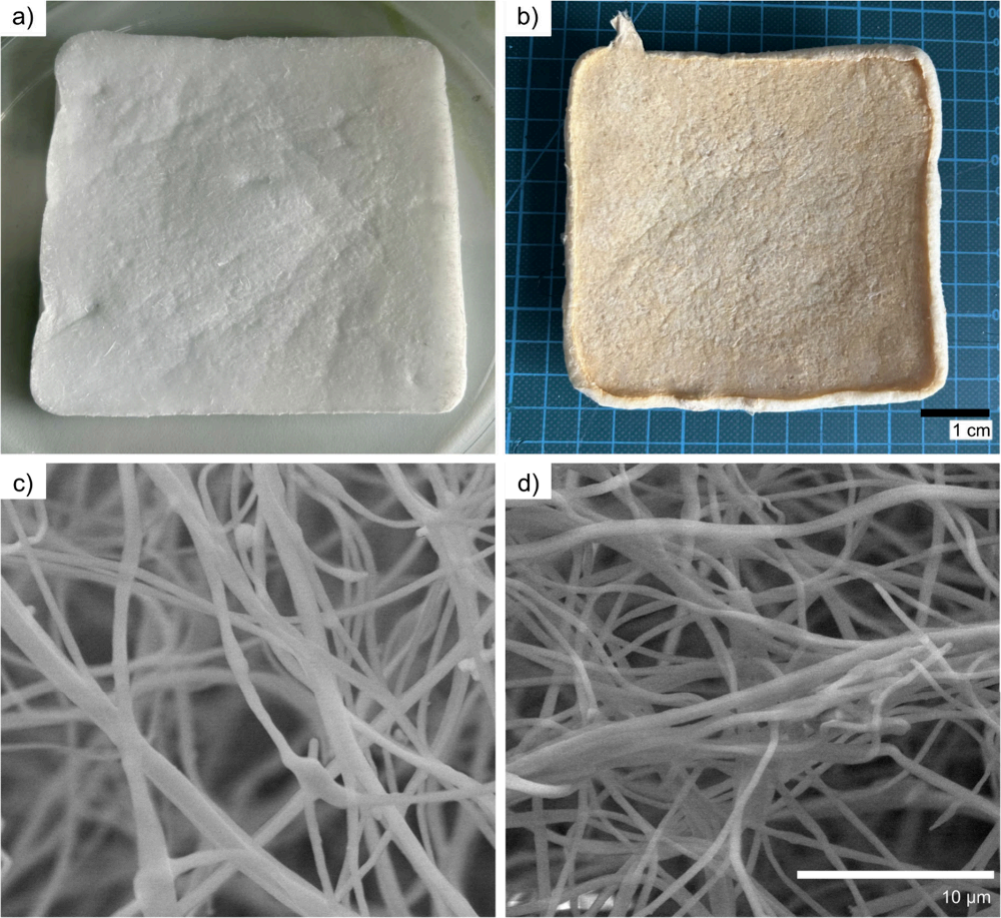
Sponge appearance (a) before cross-linking and (b) after cross-linking and SEM images of the samples (c) without MA and (d) cross-linked with 33% MA.

The sponges exhibited a highly open structure with large pores (Figure 2c and d, Figure 3a, Figure S1, Figure S2). Nano-CT analysis provided a three-dimensional representation of the sponges (Figure 3a), which was used for estimating the porosity and fiber thickness distribution (Table 1). All sponges, considering the margin of error of the measurements (Figure S3), had approximately 90% porosity and an average fiber thickness of 0.43 μm, which falls within the range commonly reported for electrospun fibers (300 to 1000 nm).^43,44^

**Figure 3:**
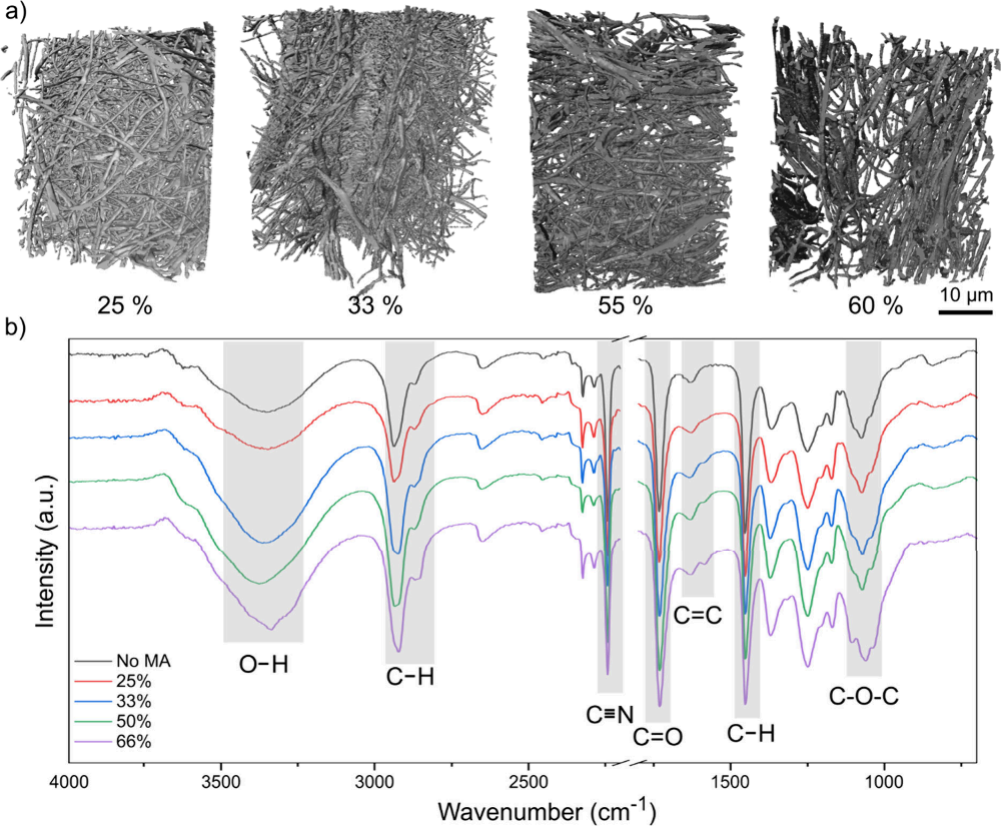
(a) Three-dimensional rendering and (b) FTIR absorption spectra of the PAN/PVA sponges cross-linked with different MA concentrations.

**Table 1:** Porosity and Fiber Thickness Estimated of all PAN/PVA Sponge Samples with Different MA Concentrations Using Three-Dimensional Nano-CT Images

MA content (%)	Porosity (%)	Fiber thickness (μm)
25	87	0.43 ± 0.01
33	92	0.42 ± 0.01
50	87	0.43 ± 0.03
66	92	0.45 ± 0.04

MA hydrolyzes in water, producing maleic acid, which serves as a cross-linker for the PVA. Given PVA’s inherent solubility in water, cross-linking is crucial for maintaining the stability of the sponges in a wet environment. Furthermore, the PVA fibers act as a binding agent, and their cross-linking plays a pivotal role in holding the PAN/PVA fibrous network skeleton together during sponge formation.^18,37^ Sponges without MA or inadequately cross-linked were unstable in water (Figure S4).

FTIR spectroscopy revealed characteristic bands between 1120 and 1000 cm^–1^ corresponding to C–O vibration of C–O–C groups,^45,46^ providing strong evidence for the cross-linking of PVA through reaction with MA (Figure 3b). The presence of other bands related to PAN and PVA, which remain unchanged, are also marked in the spectra, as well as the bands related to MA, which exhibit intensity changes with increasing MA content, such as the OH stretching band at 3300 cm^–1^ and the C=C stretching vibrations between 1570 and 1650 cm^–1^.^46–48^ An increase in the intensity of the OH stretching band for sponges with 33% and 66% MA suggests an excess of hydrolyzed MA in these samples.

The water absorption capacity of the sponges was evaluated by immersion in water for 3 h. All sponges containing 33% or more MA were fully soaked and absorbed over 100 times their initial weight (Table 2), with the highest uptake (160 times) observed in the 33% MA sample. For comparison with the maximum load, excess water was blotted off before reweighing the samples, and the same trend was observed. These blotted sample weights correspond to a reduced water content relative to fully soaked samples, as blotting removes loosely held surface water. Blotting provides an approximation rather than an exact quantification of the tightly retained residual water within the scaffolds. The 25% MA sponge was unstable and partially dissolved in water, indicating insufficient cross-linking of PVA at this MA concentration to produce stable sponges. Thus, the 33% MA sponge was selected for further testing. Its stability and degree of cross-linking were determined by Soxhlet extraction in water and gravimetry,^49^ resulting in 98.26 ± 0.02% (Table S1 and Figure S5). Critically, thermogravimetric analysis (TGA) of the cross-linked sponges under nitrogen atmosphere (Figure S6) demonstrates negligible weight loss below 100 °C, and a stable mass close to 100% up to ca. 250 °C, and indicates that physically adsorbed or capillary-bound water is effectively removed after drying and handling. The main mass loss steps observed at higher temperatures correspond to the thermal degradation of PAN and PVA components, consistent with known degradation mechanisms.^50,51^

**Table 2:** Calculated Water Uptake (WU) of Fully Soaked Sponges (WU_soaked_) and Sponges Blotted with Filter Paper (WU_blot_) after 3 h of Swelling in Pure Water

MA concentration (%)	WU_soaked_ (%)	WU_blot_ (%)
25	9015 ± 372	919 ± 49
33	16,003 ± 902	1142 ± 171
50	12,635 ± 867	1029 ± 211
66	13,422 ± 245	914 ± 75

We tested the mechanical properties of the sponge with 33% MA by performing a tensile test. The stress–strain curves obtained (Figure 4) exhibit behavior typical of foam-like materials,^52^ showing an initial quasi-linear region at low strains, followed by a peak stress corresponding to the sponge’s maximum load capacity. After this point, the sponge fails completely and the stress drops to zero. The measured Young’s modulus for the dry sponge was 994 ± 1 Pa, indicating that it undergoes large deformation under relatively low load, even compared with other soft materials.^53^ The modulus for the wet sponge was 330 ± 1 Pa, reflecting the strong effect of moisture on the mechanical behavior and the increased flexibility of the hydrated structure. The initial stress observed in Figure 4 arises from the small pre tension applied to remove slack and ensure proper alignment of the sample between the clamps. This does not influence the Young’s modulus values, which depend on the slope of the linear region. The Young’s moduli obtained are comparable to those of natural soft tissues and ultralight polymer sponges, which range from 0.1 kPa to 1 MPa.^54,55^ Notably, the sponges ruptured along a diagonal path during testing (Figure 4, red arrows), indicating that tearing initiates on one side and propagates toward the opposite extremity. This behavior suggests that stacking layers of material in an X-shaped braid could help mitigate tearing or enhance mechanical resistance.^56,57^

**Figure 4:**
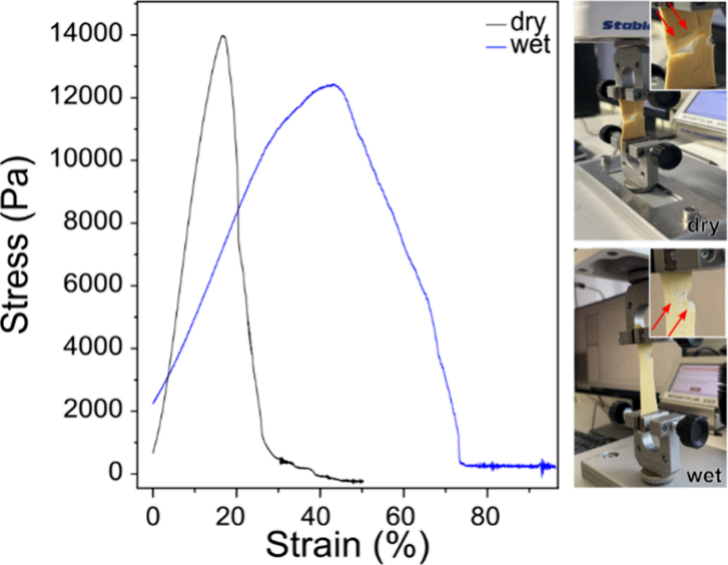
Tensile tests of sponges. Exemplary stress–strain curves for a dry and a wet PAN/PVA sponge cross-linked with 33% MA. The slope in the linear region of the curves was used for the calculation of Young’s modulus. The corresponding samples after the tensile test are shown on the right with the red arrows indicating the place of rupture.

Further tensile tests in a micro-CT device of a soaked sponge revealed its deformation characteristics (Figure 5a). Virtual slices showed slight pore displacement and deformation (Figure 5b), with the pore in the lower region moving downward (Figure 5b, blue arrows), while the pore in the middle region exhibited less displacement (Figure 5b, red arrows). Transversal slices indicated up to 36% reduction in width under stress (Figure 5c and d). This thinning could lead to sample disruption if greater forces were applied. Sample thinning was also observed in the volume fraction calculated for each data set (Figure S7).

**Figure 5:**
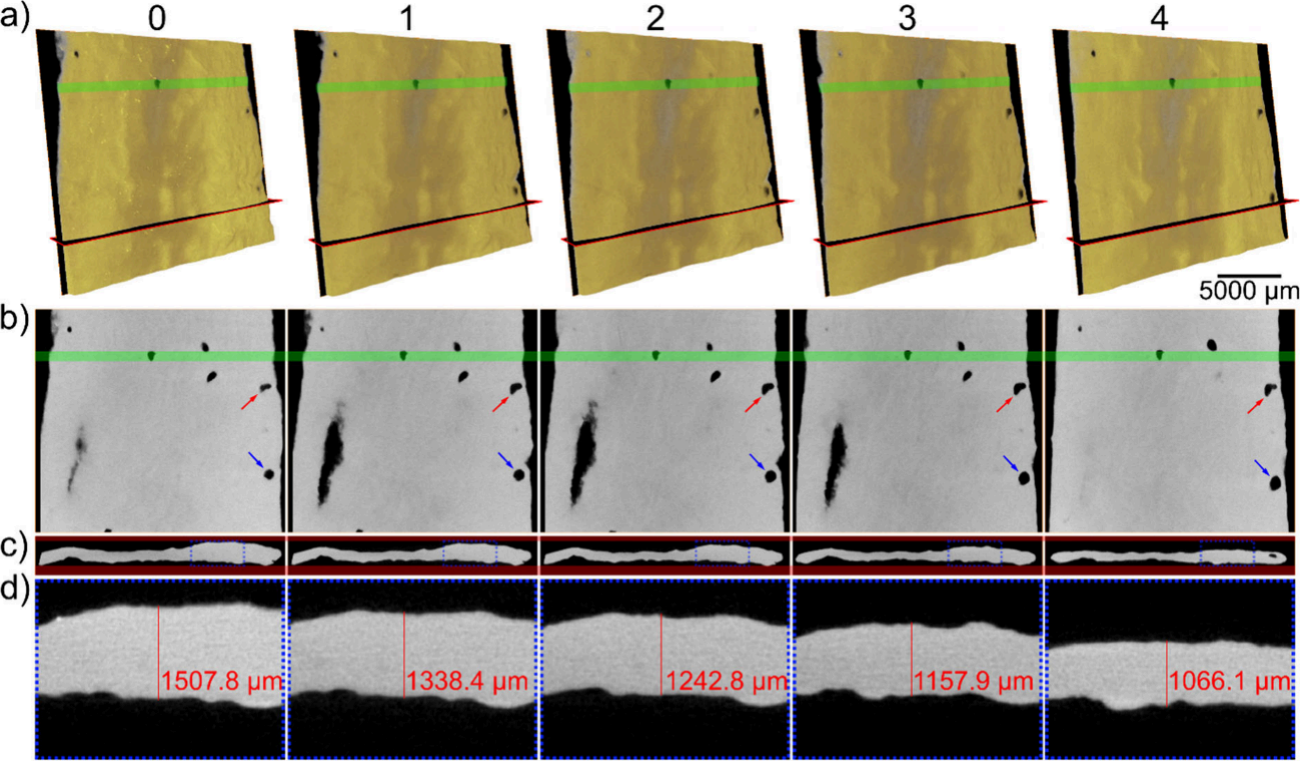
(a) Volumetric rendering of the wet pseudocolored PAN/PVA sponge at different stretching states: initial (reference) state (0) and after each extension step (1 to 4), showing one vertical and one horizontal virtual slices, (b) vertical virtual slices, with the green line marking the height of the feature used for image registration, and (c) comparison of the horizontal virtual slices. The inset area within blue dots is shown zoomed-in in (d) with an explicit measurement of the width at the same position in every scan.

We analyzed the volumetric images of the sponge during the tensile test using global Digital Volume Correlation (DVC), focusing on the initial segments of the complete stress–strain curves (Figure 6a). Our estimation of the displacement field at each stretching stage (Figure 6b) reveals an increase in displacement vectors with strain initially uniform but becoming localized with continued loading (Figure 6a, b). From the second tensile stage onward (Figure 6, stages 2–3 and 3–4), the vectors at the vertical extremes of the sample direct outward in the stretching direction, while those at the sides and middle point inward. This behavior indicates that the sample undergoes thinning more pronouncedly in one specific region as it stretches. The color strain map of the sample (detailed in Figure 6a) illustrates an increase in strain with each applied stretch. The strain map indicated a potential breaking point consistent with the rupture direction observed during the tensile tests for both dry and wet samples (Figure 4).

**Figure 6:**
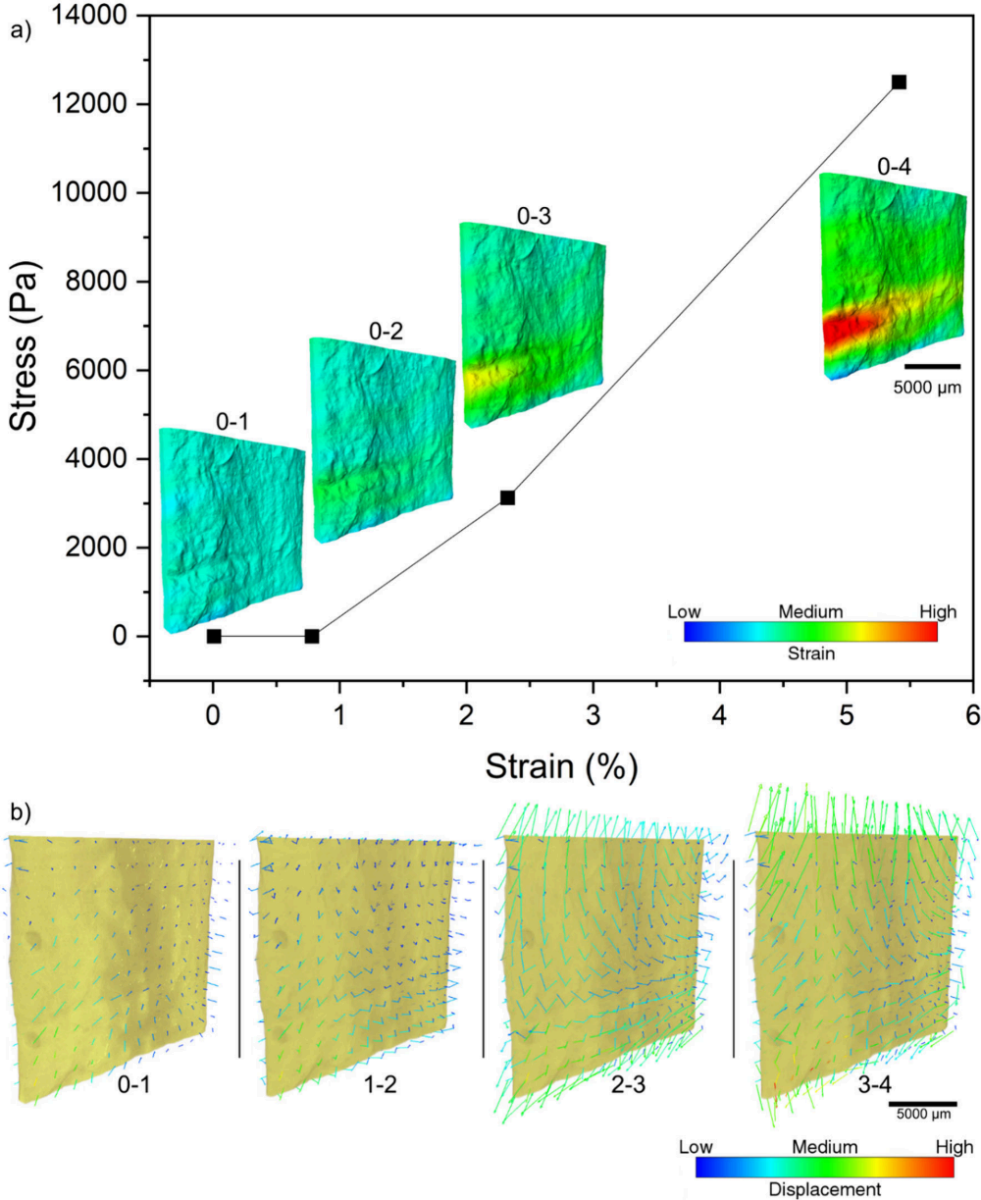
(a) Stress–strain curve of the sample imaged during *in situ* tensile testing in micro-CT. Volumetric images show the surface of the wet sponge with a standardized color field that represents the strain. In (b), the volumetric images are superimposed by the displacement vectors calculated using DVC.

## 3 Discussion

The synthesis of PAN/PVA sponges via electrospinning, short-fiber suspension preparation, freeze-drying, and subsequent MA cross-linking yielded materials with high porosity and favorable mechanical properties. Freeze-drying was specifically chosen to preserve the three-dimensional fibrous architecture of the electrospun short fibers while achieving an ultralight open-cell structure that facilitates exceptional water absorption. Although freeze-drying can reduce mechanical strength compared to alternative fabrication methods,^58^ the combined effect of PAN’s inherent rigidity and the stabilizing PVA cross-linking produced sponges with mechanical performance comparable to soft biological tissues.^53^ The observed porosity of approximately 90% highlights the highly interconnected open-pore structure of the sponges, which is advantageous for applications requiring high fluid absorption and retention. The average fiber thickness of approximately 0.4 μm aligns well with the existing literature and shows the reproducibility of the electrospinning process performed.

Our results demonstrate that the incorporation of MA is crucial for enhancing the stability of PAN/PVA sponges in aqueous environments. The water uptake capacity of the sponges highlights their potential for applications in fields such as wound healing and drug delivery, where moisture retention is essential. The sponge with 33% MA exhibited the highest water absorption capacity, confirming its stability and functionality, whereas the 25% MA sponge was unstable and partially dissolved, underscoring the need for sufficient cross-linking to prevent dissolution upon hydration.

FTIR analysis elucidated the chemical interactions within the sponges, particularly the changes in the OH band intensity with an increased MA content, consistent with effective cross-linking through esterification reactions. However, an increased OH stretching band intensity at higher MA concentrations (33% and 66%) indicates the presence of hydrolyzed MA, which introduces additional hydrophilic carboxylic acid groups into the polymer matrix. These newly formed hydrophilic groups, together with residual hydroxyl functionalities, enhance water affinity and facilitate higher swelling ratios, despite the formation of covalent cross-links that typically reduce chain mobility. Similar effects have been reported in systems cross-linked with carboxylic acid–based agents, where enhanced swelling arises from the generation of ionizable sites and reduced crystallinity that increases the network free volume.^40^

Therefore, the increase in swelling observed at higher MA contents (Table 2) can be attributed to a balance between network stabilization and the introduction of additional hydrophilic groups, leading to enhanced water uptake capacity rather than suppression of hydrophilicity. Soxhlet extraction results further confirm effective cross-linking, with a degree of reaction above 98% for the sponge prepared with 33% MA.

Compared to previous studies, our PAN/PVA sponges demonstrate an exceptionally high water absorption capacity of 16000%, significantly surpassing the 5000–5700% reported for polyimide and polylactide sponges.^42,59^ This exceptional water uptake is particularly noteworthy, as many electrospun polymer sponges are mainly designed to be hydrophobic,^15,42,60^ limiting their water absorption capabilities and their use in direct contact with the human body as wound dressings and biomedical implants, for example. Wet dressings are known to accelerate wound healing compared to dry dressings because renewed skin formation without inflammation and eschar development can only effectively occur within a moist environment, which also supports optimal cellular functions.^9^ This underscores the importance of developing highly hydrophilic and water-retentive materials, such as the present PAN/PVA sponges for advanced wound care. Although similar water absorption levels have been reported in certain hydrophilic PAN-based sponges,^30^ direct comparisons are challenging due to differences in density, porosity, and preparation methods, which likely also affect their mechanical stability. Furthermore, compared to other PVA-based sponges, the PAN/PVA sponges presented in this study outperform literature-reported water absorption capacities, which typically range from 800% to 4000%.^20,61,62^

The mechanical properties of the developed PAN/PVA sponges were characterized by Young’s moduli of approximately 1.0 kPa in the dry state and 0.33 kPa when wet. Literature reports that Young’s moduli of human skin ranges from 1 kPa to 80 MPa, depending on anatomical site, age, and testing method.^63^ This similarity supports the potential application of the sponges as wound dressings or tissue engineering scaffolds, where mechanical compliance is crucial. Although the sponges replicate the softness of skin tissue, their slightly lower stiffness in the hydrated state may influence cell attachment and integration. Future studies could address this by adjusting the cross-linking density or modifying fiber composition to tailor stiffness and extensibility for specific tissue requirements.

Our study combined in situ X-ray computed tomography with DVC to investigate the deformation and failure mechanisms of the ultraporous PAN/PVA sponges under tensile stress. To the best of our knowledge, this is the first application of in situ micro-CT tensile testing coupled with DVC on hydrated, soft polymeric sponges. The global DVC approach enabled the generation of three-dimensional maps of displacement and strain throughout the analyzed volume, quantifying how microstructural features moved and deformed under an applied load. By capturing multiple deformation steps, this method provided stepwise mappings of displacement and strain, offering mechanistic insights beyond conventional mechanical testing. The observed thinning and localized strain accumulation were key to understanding the failure process, and the correlation between strain localization and rupture direction may guide future design strategies to enhance the durability and mechanical performance of similar porous materials.

PAN has been widely utilized in biomedical and separation applications, particularly in the fabrication of hollow fiber membranes for hemodialysis and water purification.^64^ These applications benefit from PAN’s excellent mechanical strength, chemical resistance, and structural stability in aqueous environments. However, concerns remain regarding its long-term biocompatibility when used in direct contact with living tissues, owing to the potential release of degradation products.^64^ To address these challenges, several surface modification strategies have been developed, including partial hydrolysis to generate poly­(acrylic acid) groups, plasma or alkaline treatments to improve surface hydrophilicity, and grafting of biocompatible or hydrophilic polymers to enhance cellular interactions.^65,66^ Alternatively, PAN-based materials may be best suited for external or short-term biomedical applications, such as wound dressings, where prolonged implantation is not required. The incorporation of PVA into the present system further improves hydrophilicity and compatibility, offering a balanced combination of structural integrity and biological performance. Besides that, the PVA/PAN ratio employed in this study was selected based on preliminary considerations to achieve a practical balance between hydrophilicity, mechanical integrity, and processability, as demonstrated by the successful fabrication of ultraporous sponges with high water absorption capacity. While systematic optimization of this ratio was beyond the scope of the present work, future studies could explore this aspect in detail to further fine-tune the balance among mechanical robustness, hydrophilicity, and biocompatibility, thereby broadening the applicability of these ultraporous sponges.

## 4 Conclusions

In this study, ultraporous PAN/PVA sponges with 90% porosity and finely structured fibers were successfully fabricated through electrospinning, freeze-drying, and PVA cross-linking using maleic anhydride under optimized heat treatment conditions. The resulting sponges exhibited remarkable hydrophilicity, with a water absorption capacity of 16000% of their dry weight—significantly higher than that of previously reported sponges, which are typically designed for oil absorption. Mechanical characterization revealed that the wet sponge was more compliant, with a Young’s modulus approximately one-third that of the dry state, closely resembling that of natural soft tissues. Digital Volume Correlation analysis further revealed strain localization and microstructural evolution under tensile stress, providing insights into deformation and fracture behavior. While PVA is recognized for its biocompatibility, PAN may require surface modification to enhance its compatibility with biological tissues. The combination of mechanical compatibility, structural stability, and high hydrophilicity positions the PAN/PVA sponge as a promising candidate for further research and development in medical and soft material applications.

## 5 Materials and Methods

### 5.1 Synthesis of PAN/PVA Sponges

The preparation of PAN/PVA sponges consists of four fundamental steps: electrospinning; creation of a short fiber suspension; freeze-drying; and cross-linking. Electrospinning was performed on a Fluidnatek LE-50 device (Bioinicia). Reactants were used as received. The polymer solution for electrospinning was prepared by dissolving 1.4 g of PAN powder (MW 80 000, copolymer with 6% methyl acrylate, Polysciences) in 7 mL of dimethyl sulfoxide (DMSO), resulting in a concentration of 14% (w/w) and 0.2 g of PVA (Mowiol 4-88, Carl Roth) in 3 mL of DMSO (2% w/w), separately. The mixtures were mechanically stirred for 24 h to obtain homogeneous suspensions. Subsequently, the PVA solution was added to the PAN solution, and the mixture was stirred for 1 h. The mixture containing both PAN and PVA in DMSO was filled into a 20 mL syringe with a diameter of 18.5 mm, and this was connected to a uniaxial spinning head with a needle of the electrospinning device. The high voltage was set at 23 kV for the spinning head and −2 kV for the drum collector. The feed rate of the solution was fixed at 1 mL/h, and the distance between the tip of the needle and the collector drum collector, covered with one layer of polypropylene while rotating at 300 rpm was kept at 15 cm. The spinning process lasted for 5 h. The electrospun PAN/PVA mat was subsequently dried at room temperature for 24 h. To produce short fibers, 0.5 g of the electrospun PAN/PVA mat was cut into small pieces with scissors and subsequently immersed in a mixture of isopropyl alcohol and water 1:1 (125 mL each), resulting in a fiber concentration of 2 mg/mL. Next, the fibers were blended to smaller pieces with a dispersing device (T 18 digital Ultra Turrax, IKA) for 15 min until a homogeneous short fiber suspension with fiber lengths of around 200 μm was obtained. The length distribution was checked by light microscopy (Olympus BX 51). Cross-linking of PVA with maleic anhydrides (MA) was performed by dissolving MA directly into this short-fiber suspension at four different concentrations (25, 33, 50 and 66%) with respect to the PVA content. MA readily hydrolyzes to form maleic acid upon exposure to moisture, leading to the formation of maleic acid. Approximately 30 mL of the MA-containing short-fiber suspension was poured into a square mold (10 × 10 cm^2^) and kept in a fridge at −80 °C for about 24 h. Subsequently the samples were put into a freeze-dryer (Alpha 2-4 LSCplus, Christ) under 1 mbar for 24 h and afterward annealed for 1 h at 160 °C for PVA cross-linking.

### 5.2 Characterization of PAN/PVA Sponges

#### 5.2.1 Determination of Degree of Cross-Linking through Soxhlet Extraction

The sponge samples were first cut into small pieces and dried in an oven at 40 °C for 24 h to remove residual moisture prior to weighing. The initial dry mass of each sample (*m*_1_) was recorded using analytical balance precision. Soxhlet extraction was then performed for 48 h using distilled water as the extraction solvent, selected for its ability to dissolve un-cross-linked PVA while leaving the cross-linked network intact. The extraction was conducted at the boiling point of water under reflux conditions to achieve exhaustive removal of soluble components. After extraction, the samples were removed from the apparatus and dried again at 60 °C under vacuum to constant weight to eliminate any residual solvent. The final dry mass (*m*_2_) was measured. The gel content *G*, representing the degree of cross-linking, was calculated as the percentage of the insoluble fraction remaining after extraction, according to Equation 1:

1 $$G\left(Soxhlet\right)=\frac{m_{1}-m_{2}}{m_{1}}\times 100\%$$

#### 5.2.2 FTIR

PAN/PVA sponges were analyzed by Fourier-transform infrared spectroscopy (FTIR) with an Invenio R instrument (Bruker Optics). A blank spectrum in air was recorded as a reference and subtracted from all sample spectra. Measurements were taken from 100 cm^–1^ to 4500 cm^–1^ in steps of 2 cm^–1^ and a total of 64 scans was acquired. Spectra were normalized using the band at 1452 cm^–1^ (CH_2_ bending), which is not expected to change due to PVA cross-linking.

#### 5.2.3 Swelling Experiments

Cross-linked sponges were weighed and immersed in deionized water with a droplet of eosin G (0.5% (w/w) solution) only for coloring purposes. They were kept in solution for 3 h to reach swelling equilibrium. The sponges were weighed when they were fully soaked, which corresponded to the maximum load. Afterward, the excess water was removed with a piece of filter paper before the specimens were weighed again. The degree of swelling, or the water uptake *WU*, was determined by the following Equation 2:

2 $$WU=\frac{m_{wet}-m_{dry}}{m_{dry}}\times 100\%$$

where *m*_*wet*_ represents the weight in wet states, and *m*_*dry*_ represents the weight before any contact with water.^67,68^ In the following, *m*_*wet*_ refers to two different stages of wetness: one stage corresponds to the weight of the sponge when it is fully soaked with water (*m_soaked_*) and the other to the weight of the sponge after it was gently blotted with filter paper to remove excess (*m_blot_*). The swelling experiments were done in triplicate.

#### 5.2.4 Scanning Electron Microscopy (SEM)

A Quanta 3D FEG Dual Beam from FEI Co. (Hilsboro, OH, USA) was used for Scanning Electron Microscopy (SEM) imaging. The samples were immobilized on SEM stubs by using carbon tape. No coating was applied to prevent compromising the fiber morphology. The images were recorded under high vacuum conditions, an acceleration voltage of 10 kV and a working distance of ca. 10 mm.

#### 5.2.5 X-ray Microscopy (nano-CT)

For all nano-CT experiments, thin samples ranging from one to two mm in size cut with scissors were affixed to the tip of a standard metal pin using glue. Care was taken to ensure that the samples were sufficiently large so that the glue did not penetrate the area where imaging was performed. Imaging of the samples was conducted using a Carl Zeiss Xradia 810 Ultra equipped with a rotating chromium anode to generate X-ray photons with an energy of 5.4 keV. The samples were inserted into the sample holder of the device, and for each imaging experiment, a total of 1001 projections of 10 s each were obtained with the sample rotating from −90° to +90° and a detector binning of 2 (corresponding to pixel size 128 nm). The imaging experiments were done using Zernike phase-contrast mode and a field of view of 65 μm × 65 μm. The nano-CT projection images were reconstructed using the filtered back projection algorithm in the Scout and Scan Reconstructor (version 13) software. The reconstructed volumetric images with a voxel size of 128 nm were exported as a stack of 16-bit TIFF images (tagged image file format) for subsequent analysis in Avizo software (Thermo Fisher Scientific, version 3D 2023.1). The image processing steps are detailed in the Supporting Information (Figure S9). Porosity and fiber thickness distributions for the PAN/PVA sponges were obtained.

### 5.3 Mechanical Testing

#### 5.3.1 Texture Analyzer

Mechanical tensile tests were performed on a Texture Analyzer (TA.XTplus, Stable Micro Systems) calibrated before use. Six rectangular specimens of a sponge cross-linked with 33% MA were secured with metallic clamps and tested either in the dry state (three specimens) or in water for 3 h before the test (three specimens). Tensile tests were conducted in triplicate at room temperature at 0.5 mm × s^–1^. Young’s modulus was determined from the slope of the linear portion of the stress–strain curves, serving as an indicator of the material’s stiffness. This value represents the amount of load required to induce a 1% strain in the material’s length.^53^

#### 5.3.2 *In Situ* Intermittent Tensile Testing in a Micro-CT

*In situ* deformation experiments were performed in a RayScan 200E micro-CT scanner (RayScan Technologies GmbH, Germany) using a Deben CT5000 loadcell tensile stage (Deben, UK). A sample of 6 cm × 2 cm was fixed in the tensile stage using two metallic clamps. Due to the poor X-ray absorption contrast in the PAN/PVA sponge, a 1% I_2_ with KI aqueous solution (Lugol) was prepared, and the sponge was soaked in it before the scans. The micro-CT scanner is equipped with a tungsten anode. A voltage of 70 kV and a current of 270 μA were applied, and a total of 1350 projections of 2000 ms each along 360° were obtained. After reconstruction was performed with the software provided by the device, the final images have a voxel size of 17.5 μm. Besides the reference scan without an applied load, four intermittent CT imaging experiments were conducted on the same sample to track deformation, each time doubling the extension. The extensions used were 0.158 0.265, 0.530, and 1.06 mm. After scanning, the data were analyzed using Avizo software, where a DVC global approach was employed to assess the strain map and the displacement field of the sample at each stretching stage (Figure S10).

## Supplementary Material



## Notes

Figure S1: Light-microscopy images of PAN/PVA fibers with different MA concentrations. Figure S2: Comparison of dense and open regions within the 33% MA sponge. Figure S3: Nano-CT fiber diameter distributions. Figure S4: Morphology of sponges produced without MA before and after water exposure. Table S1: Mass before/after Soxhlet extraction and calculated cross-linking degree. Figure S5: FTIR spectra before and after Soxhlet extraction. Figure S6: TGA curves of PAN/PVA sponge before and after Soxhlet extraction. Figure S7: Volume-fraction changes per slice during incremental tensile loading. Figure S8: Raman spectra of non-cross-linked and cross-linked PAN/PVA sponge. Figure S9: Nano-CT image-processing workflow. Figure S10: Micro-CT and DVC analysis workflow (PDF)
