# Supplementary material for: Engineering Ultraporous and Highly Stable Polyacrylonitrile/Poly(vinyl alcohol) Sponges with High Water Absorption Capacity
Source: ACS Appl Mater Interfaces. 2025 Dec 16;17(52):70899–907. doi: 10.1021/acsami.5c18694 (PMC12766677; doi:10.1021/acsami.5c18694)
Supplement: Supplementary file 1 [file am5c18694_si_001.pdf]

Supporting Information

Engineering Ultraporous and Highly Stable Polyacrylonitrile/Poly(vinyl alcohol) Sponges with High Water Absorption Capacity

Michèle-Louise Regner[1,2], Mateus Gruener Lima[2,3], Annika Thormann[2], Rafaela Debastiani[4,5], Juliana Martins de Souza e Silva[1,2,*]

[1] Institute of Physics, Martin Luther University Halle-Wittenberg, 06120 Halle (Saale), Germany

[2] Fraunhofer Institute for Microstructure of Materials and Systems IMWS, 06120 Halle (Saale), Germany

[3] Applied Nuclear Physics Research Group, State University of Londrina, 86057-970 Londrina, Brazil

[4] Institute of Nanotechnology (INT), Karlsruhe Institute of Technology (KIT), 76344 Eggenstein-Leopoldshafen, Germany

[5] Karlsruhe Nano Micro Facility (KNMFi), Karlsruhe Institute of Technology (KIT), 76344 Eggenstein-Leopoldshafen, Germany

*juliana.martins@physik.uni-halle.de

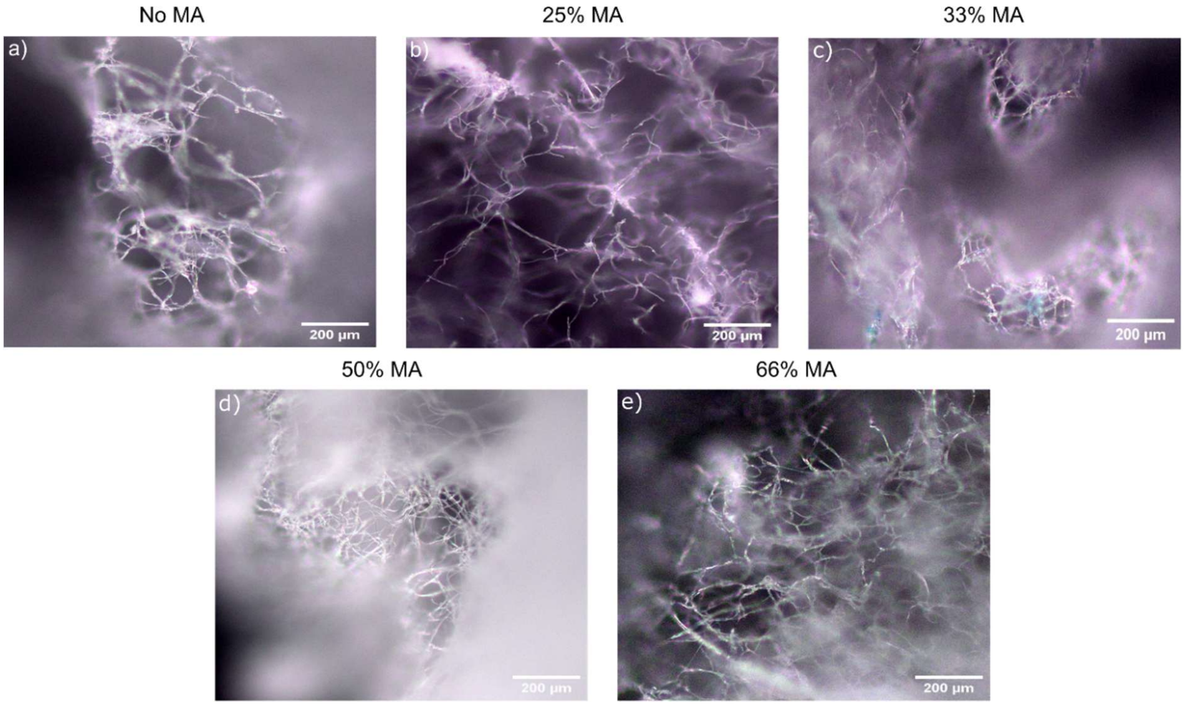

Fig. S1. Light-microscopy images of the PAN/PVA fibers with different maleic anhydride (MA) concentrations. a) 0%, b) 25%, c) 33%, d) 50%, and e) 66%.

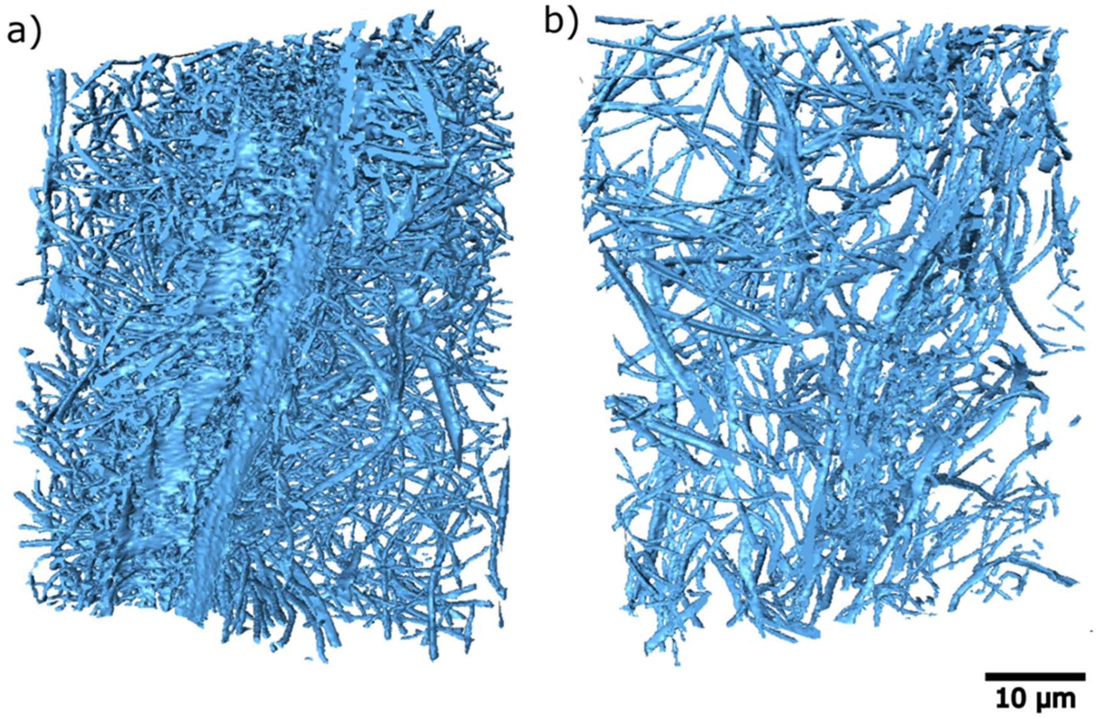

Fig. S2. Comparison of a denser and a more open structure within the same sample (33 % MA). a) Region with a lot of fibers. b) Region with larger pores.

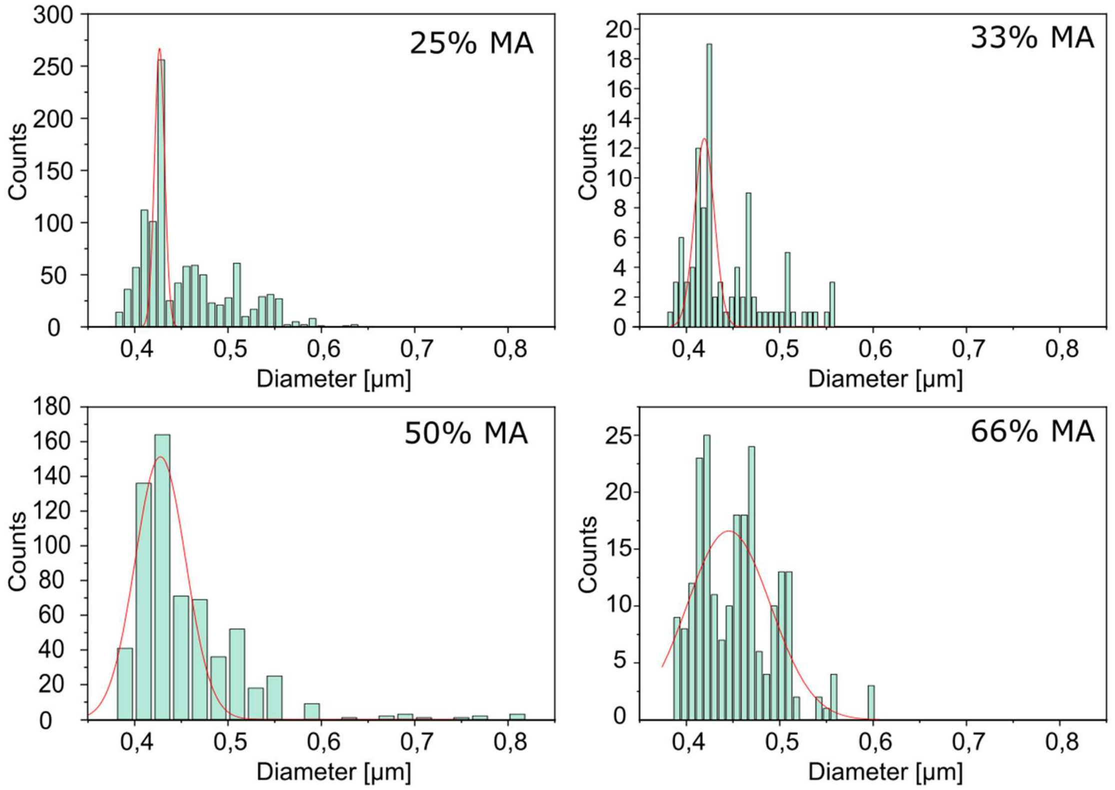

Fig. S3. Fiber diameter distribution for the different samples assessed through nano-CT. The average diameters and standard deviations were obtained using a Gaussian fit. All values below three times the voxel size were excluded from the analysis, as these were considered noise, which also significantly reduced the number of counts. This thresholding may cause a slight overestimation of the average fiber diameter, but it ensures that only reliable measurements above the resolution limit are considered.

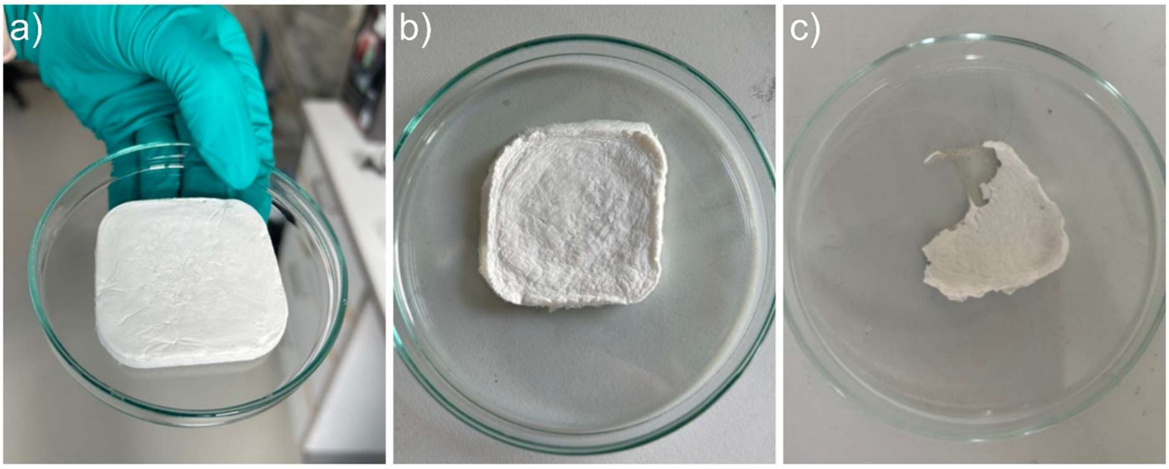

Fig. S4. Sponge made without MA a) after freeze-drying, b) after cross-linking, c) after collapse due to being in contact with water.

Soxhlet extraction

Table S1. Mass of PAN/PVA sponge specimens before and after Soxhlet extraction with water, and corresponding cross-linking degree.

Specimen	Mass before Soxhlet (mg)	Mass after Soxhlet (mg)	Cross-linking degree (%)
PAN_PVA_1	85.1	83.6	98.24
PAN_PVA_2	190.4	187.1	98.27

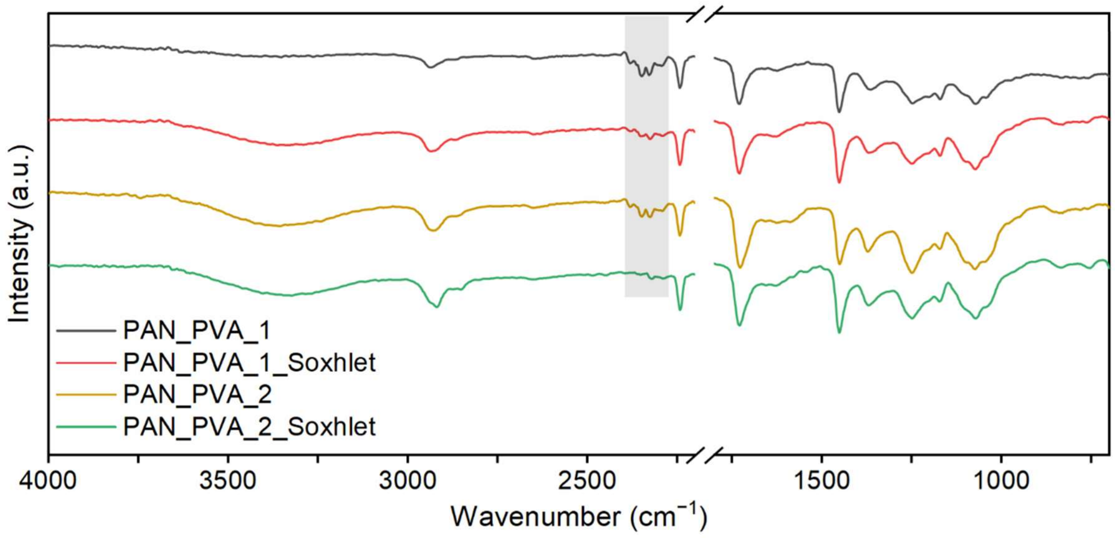

Fig. S5. FTIR absorption spectra of PAN/PVA sponge specimens cross-linked with 33 % maleic acid (MA), shown before and after Soxhlet extraction (indicated by "*_Soxhlet*").

The FTIR spectra indicate that the overall chemical structure of the PAN/PVA sponges remained largely unchanged following Soxhlet extraction, confirming the stability and effectiveness of the cross-linking with maleic acid. Most characteristic absorption bands associated with PAN and PVA (previously identified in Fig. 3) showed minimal or no significant variation after extraction.

A minor change was observed around the 2300 cm^{-1} region (highlighted in gray in Fig. S5). However, this band is commonly attributed to atmospheric carbon dioxide (CO_2) rather than to functional groups in the sponge components. Therefore, the observed difference is likely due to slight variations in background absorption during spectral acquisition, rather than any chemical modification of the material.

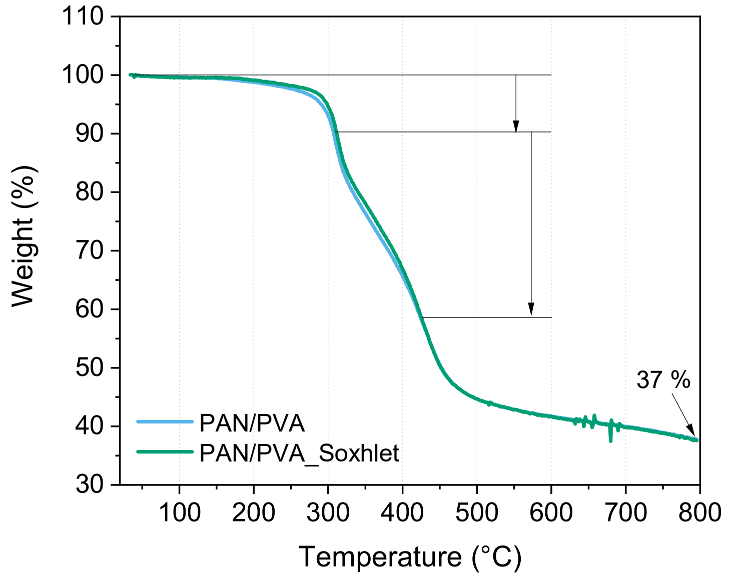

Fig. S6. TGA curves of PAN/PVA sponge before and after Soxhlet extraction in nitrogen atmosphere at 10 °C/min.

The TGA curves under nitrogen atmosphere shows no detectable weight loss below 100 °C, with sample mass remaining near 100 % up to about 250 °C. This absence of early mass loss supports negligible levels of physically adsorbed or capillary-held water inside the crosslinked PAN/PVA scaffolds after drying and handling. Significant mass loss starts only above 250 °C, well above the range expected for water evaporation, confirming that any residual water content in the analyzed samples is minimal. The main decomposition profile reveals two steps: the first (9% weight loss) between 250 °C and 350 °C, and the second (32%) between 350 °C and 500 °C. By comparison to literature TGA studies for PAN[1], and for PVA[2], the first step is attributed primarily to decomposition and cyclization reactions of PAN, while the second is mainly associated with the dehydration and chain scission of PVA, as well as further cyclization and aromatization events. Above 500 °C, ongoing mass loss reflects continued cyclization and carbonization, with the curve not reaching a plateau at 800 °C, in line with the formation of not yet fully stabilized carbonaceous residues of the cyclization and dehydrogenation of PAN chains.

[1] Cipriani et al., 2016, *Polym. Degrad. Stab.*
[2] Peng & Kong, 2007, *Polym. Degrad. Stab.*

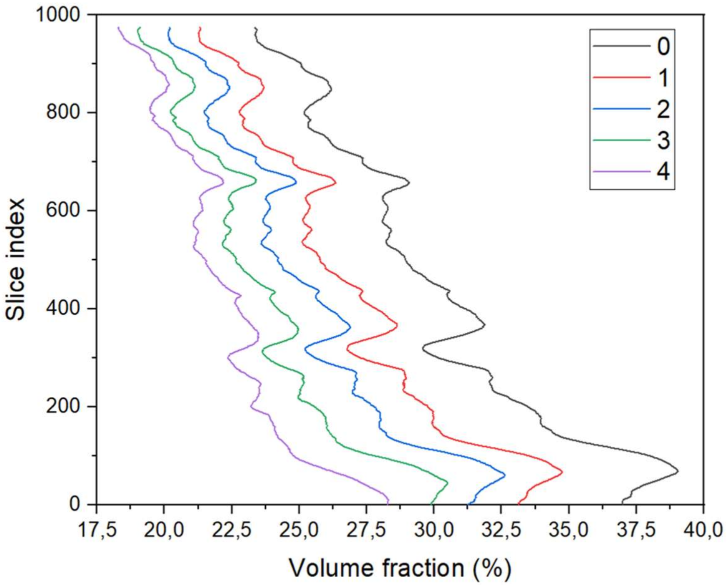

Fig. S7. Change of volume fraction for each virtual slice of a sponge after successive tensile strain steps (0: undeformed; to successive deformed states: 1-4). Slice Index: 0 corresponds to the bottom of the sample and 962 to top. Slice thickness: 17.5 µm.

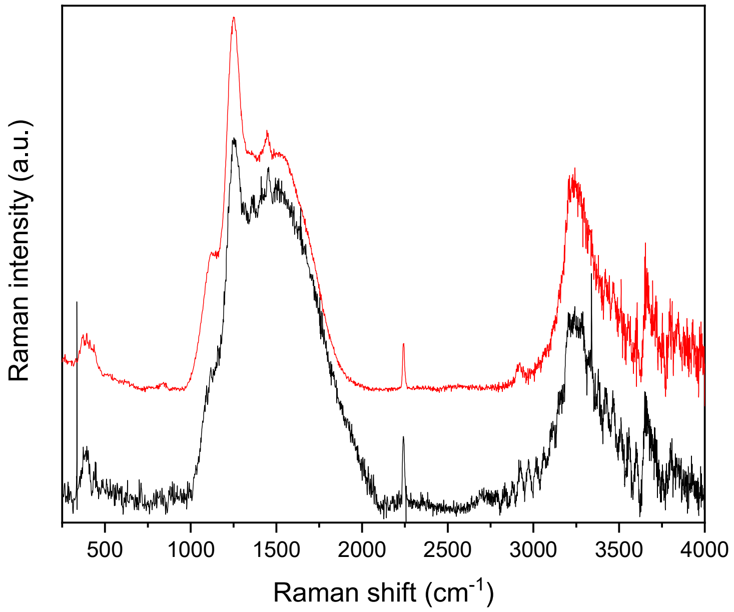

Fig. S8. Raman spectra of non-cross-linked (black) and cross-linked (red) PAN/PVA sponge sample with 33 % of MA. Raman spectroscopy was performed in a FluoroLog-3 (Horiba Jobin Yvon) spectra were acquired at 785 nm excitation wavelength, with a 100 pinhole, 2 accumulations and an objective of 50 ×.

PAN/PVA sponge containing 33 % MA was measured before and after 1 h of cross-linking. The two samples have different densities, so comparisons between samples should be made only in terms of peak position, asymmetries and intensity ratio, not in terms of intensity or area. The broad band centered at 3200 cm^{-1} corresponds to C-H symmetric and antisymmetric stretching vibrations and is the most intense Raman signal observed in PVA, and the one at 1123 cm^{-1} is due to C-O stretching. The signal at 2240 cm^{-1} is related to PAN (υ-C≡N). Electrospun PAN fibres without any treatment show an almost featureless Raman spectrum in the region between 1000 and 2000 cm^{-1}. The signals observed in this region suggest that the polymers in the sponges may have some degree of ordered arrangement.

Nano-CT data analysis

After loading the datasets in the Avizo software (Thermo Fisher Scientific, version 3D 2023.1), a volume of interest was cropped using the *Extract Subvolume* module (Fig. S9 a and b). In the following, a median filter was applied (Fig. S9 c) to reduce noise and preserve edges. Binarization of the data stack was done visually by the *Interactive Thresholding* module (Fig. S9 d), to isolate the fibers. To fill holes inside of the fibers and remove small spots (noise) smaller than 20 pixels in the images, the *Fill Holes* (Fig. S9 e) and *Remove Small Spots* (Fig. S9 f) modules were attached, respectively. The *Auto Skeleton* module first calculates a distance map of the binarized image and then performs a thinning of the fiber voxels such that a line of connected voxel remains. Furthermore, it calculates the local radius, which is the distance of the middle voxel to the nearest fiber boundary. A *Spatial Graph* object shows the 3D fiber thickness distribution of the sample. The saved data can then be exported to create a histogram of the fiber diameter distribution. For calculating the porosity of the sponges, the segmented and binarized data was inverted. The porosity of the samples was then calculated using the *Volume Fraction* module. This estimates the ratio of pixels related to the fibers to the total number of pixels in the analyzed volume, with an uncertainty of ±2 % dominated by limitations related to the resolution (isotropic pixel size of 128 nm).

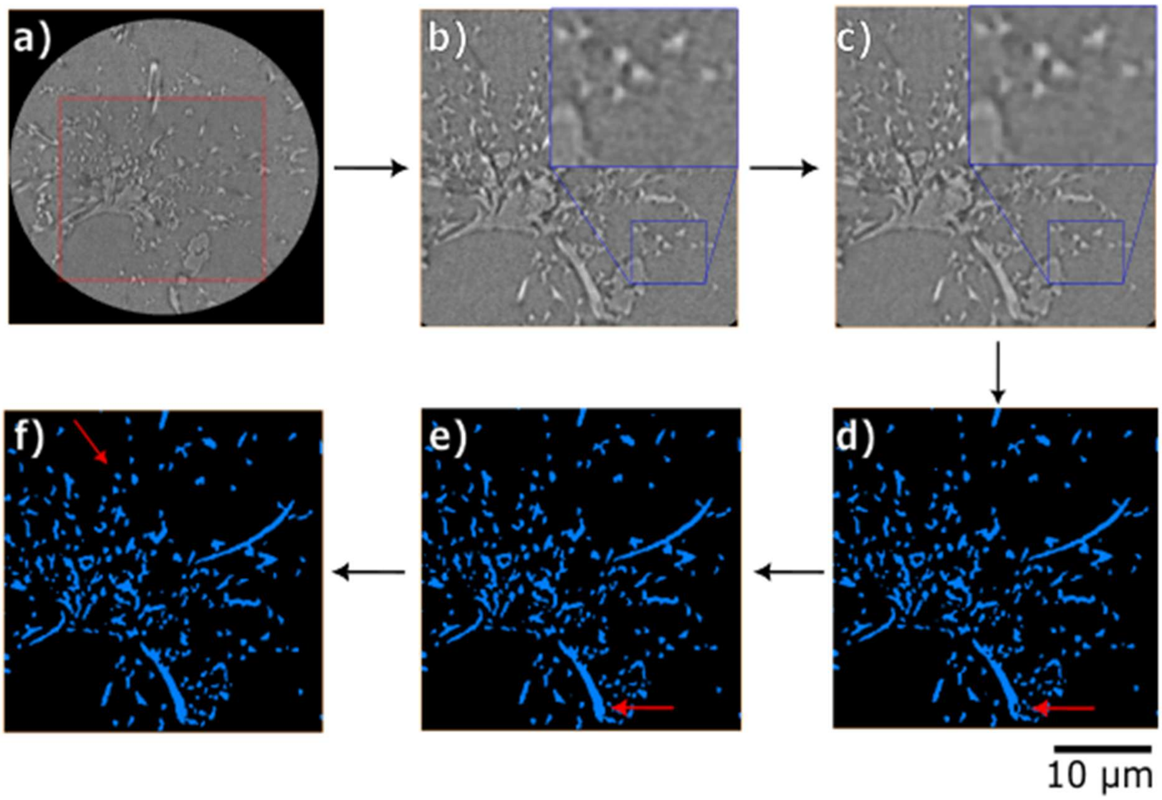

Fig. S9. Workflow for image processing for all nano-CT datasets represented by the same tomogram. a) Imported data, with chosen subvolume (red square), b) extracted subvolume, c) filtered, d) binarized image, e) holes were filled, f) small spots were removed. Red arrows point to holes and small spots that were filled or removed.

Micro-CT data and DVC analyses

The reconstructed tomograms were imported all together in Avizo. As a first step, all datasets were registered manually using a pore visible in all datasets (Fig. S10 a, red arrow). To remove the signal from the metallic two clamps used to fix the sample, a subvolume of 1090 × 170 × 962 voxels was extracted for further analyses. An *Autothreshold* (IsoData) was then applied to all datasets and the *Volume Fraction* was calculated for each slice of each dataset.

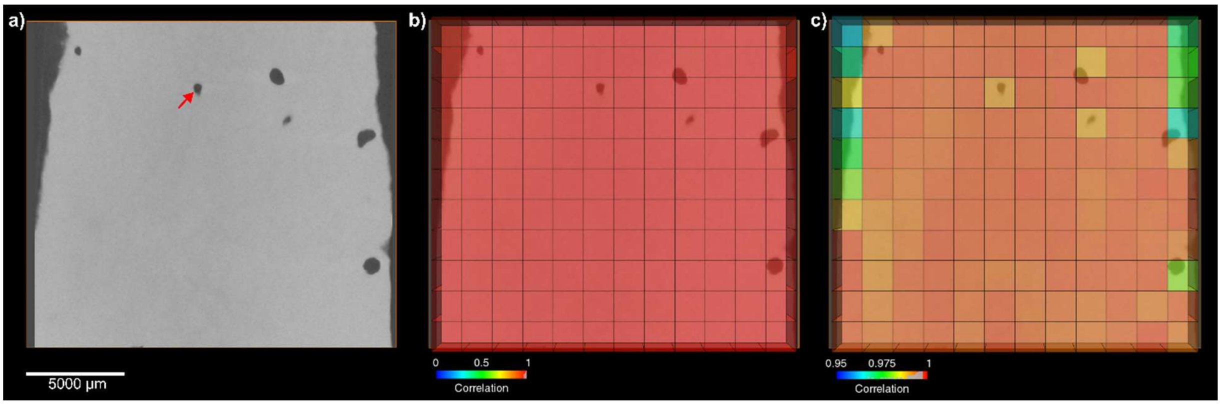

Fig. S10. a) Virtual vertical orthoslice, b) Grid view of the metric/correlation values obtained for the local approach DVC analysis with a cell size of 1475 µm, c) Correlation color map ranging from 0.95 to 1.00 (excellent values according to Thermo Fisher Scientific Avizo Software User's Guide[3]).

A subset-based (mesh) approach was used for the DVC analysis. To measure the displacement and strain maps, at least two datasets and a mesh are required. To find the best subset (cell) size, a *Radial Autocorrelation* module was applied to the first (reference) dataset. The value at which the autocorrelation function has an inflection point is called the microstructure range. According to the Thermo Fisher Scientific Avizo Software User's Guide, the optimal cell size is 3-4 times the distance at which the autocorrelation function turns into an asymptote. This value serves as an initial guess for the subset size. A *DVC Local Approach* module was attached to the datasets. The local (subset-based) approach finds iteratively the parameters of a transformation matrix that matches the best between the grey level intensity of the initial and the first deformed dataset. A metric of 1 indicates a perfect match and a metric of 0 defines no match (Fig. S10 b, and c). The subset (also called cell) values and the corresponding correlation values are displayed with a color scale. For the chosen cell size of 1475 µm, the correlation metric gave values over 0.95, which represents a good correlation. The metric is the lowest (0.95) at the sides of the sample (Fig. S10 c), which are the regions where the displacement is high. Thus, a subset size of 1475 µm was used in the *Generate Global DVC Mesh* module and an *Incremental Global DVC* could be performed.

Images in the main manuscript show displacement and strain fields based on calculations on the z-component of the displacement vector as well as the e33 (Akk) component of the Youngs' modulus for each cell.

[3] Thermo Fisher Scientific. Thermo Scientific Avizo Software 9 User's Guide, 2018.
